# Functional measures as potential indicators of down‐the‐drain chemical stress in freshwater ecological risk assessment

**DOI:** 10.1002/ieam.4568

**Published:** 2022-01-18

**Authors:** Laura J. Harrison, Katie A. Pearson, Christopher J. Wheatley, Jane K. Hill, Lorraine Maltby, Claudia Rivetti, Lucy Speirs, Piran C. L. White

**Affiliations:** ^1^ Department of Environment and Geography University of York York Heslington UK; ^2^ Department of Biology Leverhulme Centre for Anthropocene Biodiversity, University of York York Heslington UK; ^3^ School of Biosciences, The University of Sheffield Sheffield Western Bank UK; ^4^ Safety and Environmental Assurance Centre, Unilever, Colworth Science Park Sharnbrook UK

**Keywords:** Ecosystem function, Ecosystem processes, ERA, Freshwater, Functional traits

## Abstract

Conventional ecological risk assessment (ERA) predominately evaluates the impact of individual chemical stressors on a limited range of taxa, which are assumed to act as proxies to predict impacts on freshwater ecosystem function. However, it is recognized that this approach has limited ecological relevance. We reviewed the published literature to identify measures that are potential functional indicators of down‐the‐drain chemical stress, as an approach to building more ecological relevance into ERA. We found wide variation in the use of the term “ecosystem function,” and concluded it is important to distinguish between measures of processes and measures of the capacity for processes (i.e., species' functional traits). Here, we present a classification of potential functional indicators and suggest that including indicators more directly connected with processes will improve the detection of impacts on ecosystem functioning. The rate of leaf litter breakdown, oxygen production, carbon dioxide consumption, and biomass production have great potential to be used as functional indicators. However, the limited supporting evidence means that further study is needed before these measures can be fully implemented and interpreted within an ERA and regulatory context. Sensitivity to chemical stress is likely to vary among functional indicators depending on the stressor and ecosystem context. Therefore, we recommend that ERA incorporates a variety of indicators relevant to each aspect of the function of interest, such as a direct measure of a process (e.g., rate of leaf litter breakdown) and a capacity for a process (e.g., functional composition of macroinvertebrates), alongside structural indicators (e.g., taxonomic diversity of macroinvertebrates). Overall, we believe that the consideration of functional indicators can add value to ERA by providing greater ecological relevance, particularly in relation to indirect effects, functional compensation (Box 1), interactions of multiple stressors, and the importance of ecosystem context. *Environ Assess Manag* 2022;18:1135–1147. © 2022 The Authors. *Integrated Environmental Assessment and Management* published by Wiley Periodicals LLC on behalf of Society of Environmental Toxicology & Chemistry (SETAC).

## Key Points

- Considering functional indicators in addition to structural indicators adds value to ERA, but wide variation in the use of the term "ecosystem function" limits implementation.
- We classify different types of potential functional indicators and argue it is important to distinguish between measures of processes and measures of the capacity for processes (i.e., species' functional traits).
- Some measures have great potential to be developed as functional indicators (such as rate of leaf litter breakdown, oxygen production, carbon dioxide consumption, and biomass production), but further study is needed before these measures can be implemented and interpreted within a regulatory context.
- It is valuable for ERA to include multiple types of indicators relevant to each function of interest (e.g., both rate of leaf litter breakdown and functional composition of macroinvertebrates), alongside structural indicators.

## INTRODUCTION

Chemical stressors and their mixtures may have adverse effects on freshwater ecosystems, causing potential impacts on their components (biotic and abiotic structure, including biodiversity) and operation (processes and functions; Borgwardt et al., 2019; Peters et al., 2013; Sandin & Solimini, 2009; Woodward et al., 2012). Concerns about understanding how chemical stressors may affect freshwater ecosystems have been especially directed toward urban effluents, which are complex mixtures, including primarily sewage and down‐the‐drain chemicals. This latter group comprises a wide range of different chemical classes such as pharmaceuticals (Petrie et al., 2015; Rosi‐Marshall & Royer, 2012), personal care and household products (Chaves et al., 2020), and nanomaterials (Miao, Guo, et al., 2019; Zhai et al., 2018) that are released in various temporal and spatial patterns, including being treated in sewage treatment plants or through direct exposure. This poses a complex challenge to the assessment of their impact on freshwater ecosystems (Borgwardt et al., 2019). Aquatic ecological risk assessment (ERA) is an accepted regulatory framework to estimate the likelihood and associated uncertainty that individual chemical stressors may cause adverse ecological effects on assessment endpoints (Box 1), by using taxonomically representative species from three trophic levels (European Chemicals Bureau, 2003). Conventional ERA assumes that defining conservative stressor thresholds based on individual species sensitivity will protect the overall ecosystem, including its processes and functions (McCormick et al., 1991; Versteeg et al., 1999).

Box 1 **GLOSSARY**

*Assessment endpoints*: Ecological entities and their attributes upon which effects of exposure to a stressor are assessed within ERA, for example, organism growth or population abundance.

*Ecosystem services*: Conversion of ecosystem functions into a service to society (de Groot et al., 2002) that underpins benefits to humans (Raffaelli & White, 2013). For example, water purification is a service resulting from nutrient cycling and organic matter transformation, which, in combination with other capital inputs (e.g., financial, labor), produces goods such as clean drinking water.

*Ecosystem structure*: The abundance, distribution, and interaction of all the living (biotic) components (studied at the levels of genotype, phenotype, population, species, and community) and the nonliving (abiotic) components (such as nutrients and other physical habitat features) that constitute an ecosystem.

*Ecosystem processes*: Interaction between biotic and abiotic components of the ecosystem (de Groot et al., 2002; von Schiller et al., 2017), for example, organic matter accumulation.

*Ecosystem function*: The net effect of processes that control fluxes of energy and matter through ecosystems, linking the various structural components of the ecosystem (Jax, 2005; Odum, 1962), for example, productivity or nutrient cycling.

*Functional compensation*: The replacement of one species' (or taxon's) contribution to an ecosystem function by another species (or taxon), when the first species (or taxon) is impaired, declines, or goes extinct (Rosenfeld, 2002).

*Functional effect trait*: The behavioral, biochemical, morphological, physiological, or phenological characteristics of organisms that underlie the impact of an organism on ecosystem properties and processes (Díaz et al., 2013).

However, the limited ecological relevance of this approach has been recognized (Cairns, 1986; Galic et al., 2017; Maltby et al., 2018; Sandin & Solimini, 2009; Van den Brink, 2008), with evidence that impacts on ecosystem functions can occur below regulatory thresholds set according to impacts on ecosystem structure alone (Box 1; Peters et al., 2013). Ecosystem functions, such as primary productivity or organic matter transformation, are defined as the net effect of processes that control fluxes of energy and matter through ecosystems (de Groot et al., 2002; Farnsworth et al., 2017; Odum, 1962; von Schiller et al., 2017). For example, the processes of leaching, physical abrasion, and decomposition by bacteria, fungi, and invertebrates all contribute to the function of organic matter transformation in aquatic ecosystems, with the resulting maintenance of soil and water quality (ecosystem services). Hence, preserving ecosystem functions is of particular concern because they ensure the safe operating space of ecosystems is maintained and directly underpin ecosystem services that support human well‐being (Bruins et al., 2017; Díaz et al., 2013).

For this reason, there is a pressing need to determine additional endpoints relevant to ecosystem functions, as well as methods for measuring the effects of exposure to chemical stressors on these endpoints for their implementation within ERA. Because it is particularly difficult to measure ecosystem functions directly (Lamont, 1995), indicators are sought that either directly measure an attribute of a contributing process or have an indirect but causal relationship with the function (Lindenmayer & Likens, 2011). In common with all good indicators, these functional indicators need to be easily applicable and transferable between contexts, with sufficiently sensitive and consistent responses to chemical stress to provide meaningful information for decision‐making within an ERA (Kurtz et al., 2001; Niemi & McDonald, 2004).

Functional indicators can be applied within ERA for a wide variety of chemical stressors and release routes. Here, we particularly consider how functional measures have been used in contexts where down‐the‐drain chemical stress is present, to highlight different types of potential functional indicators and points to consider during their development. We review the literature to:

i. Provide an overview of potential indicators of ecosystem function, based on measures used in studies of freshwater ecosystems where down‐the‐drain chemical stress is present;
ii. Classify these potential functional indicators into categories according to the different aspects of ecosystem processes they measure that contribute to functions (Box 1);
iii. Consider how potential functional indicators score against good indicator criteria and which functional indicators have the greatest potential to increase the ecological relevance of ERA.

## METHODS

We searched the Web of Science database on 22 February 2021 using both specific and broad search terms to capture literature that included functional measures in freshwater ecosystems in the presence of down‐the‐drain chemicals:

(Freshwater OR River OR Stream OR Lake)

AND

(Biodiversity OR Richness OR Evenness OR “Biological diversity” OR “Functional diversity” OR “Ecosystem function” OR “Ecosystem Process” OR “nutrient cycling” OR “organic matter transformation” OR “primary productivity” OR “secondary productivity”)

AND

(chemical OR “personal care product” OR “household product” OR surfactant OR antimicrobial OR fragrance OR pharmaceutical OR disinfectant OR microplastic OR preservative OR “down‐the‐drain”)

Timespan: 1900–2020. Indices included were the: Science Citation Index Expanded (1900–2020), Social Sciences Citation Index (1956–2020), Arts and Humanities Citation Index (1975–2020), Conference Proceedings Citation Index—Science (1990–2020), Conference Proceedings Citation Index—Social Science & Humanities (1990–2020), and Emerging Sources Citation Index (2015–2020).

**Figure 1:**
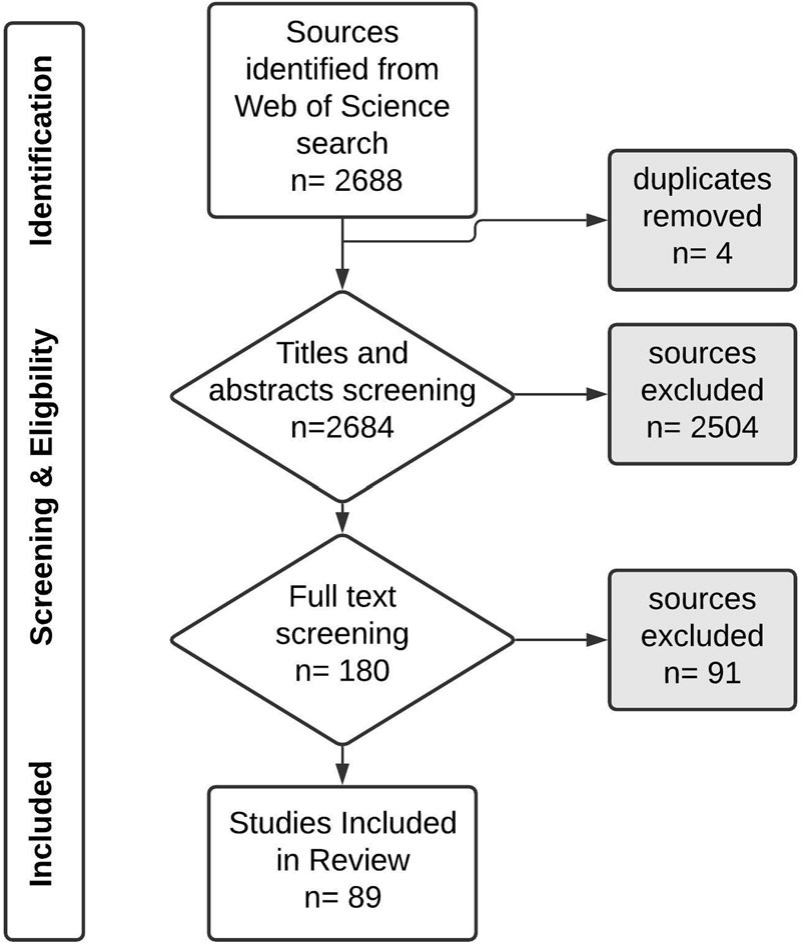
Flowchart detailing the search and selection process applied during the review. *n*, number of sources

This search returned 2688 articles on aquatic ecosystems with abstracts written in English (Figure 1 and Supporting Information figures). Titles and abstracts followed by full text were screened and then selected for inclusion where: (i) the study was conducted in a freshwater ecosystem, mesocosm, or microcosm (estuarine systems were excluded); (ii) there was a spatial or temporal presence and absence, or gradient, of down‐the‐drain chemical stress; and (iii) an ecosystem state or rate was quantified that either had the potential to be a functional indicator or was being used as a functional indicator by the authors. These selection criteria meant that studies were included if they mentioned spatial or temporal differences in down‐the‐drain chemical stress, such as the presence of a wastewater treatment plant or a difference in wastewater and/or land runoff source such as between urban and seminatural land uses.

**Figure 2:**
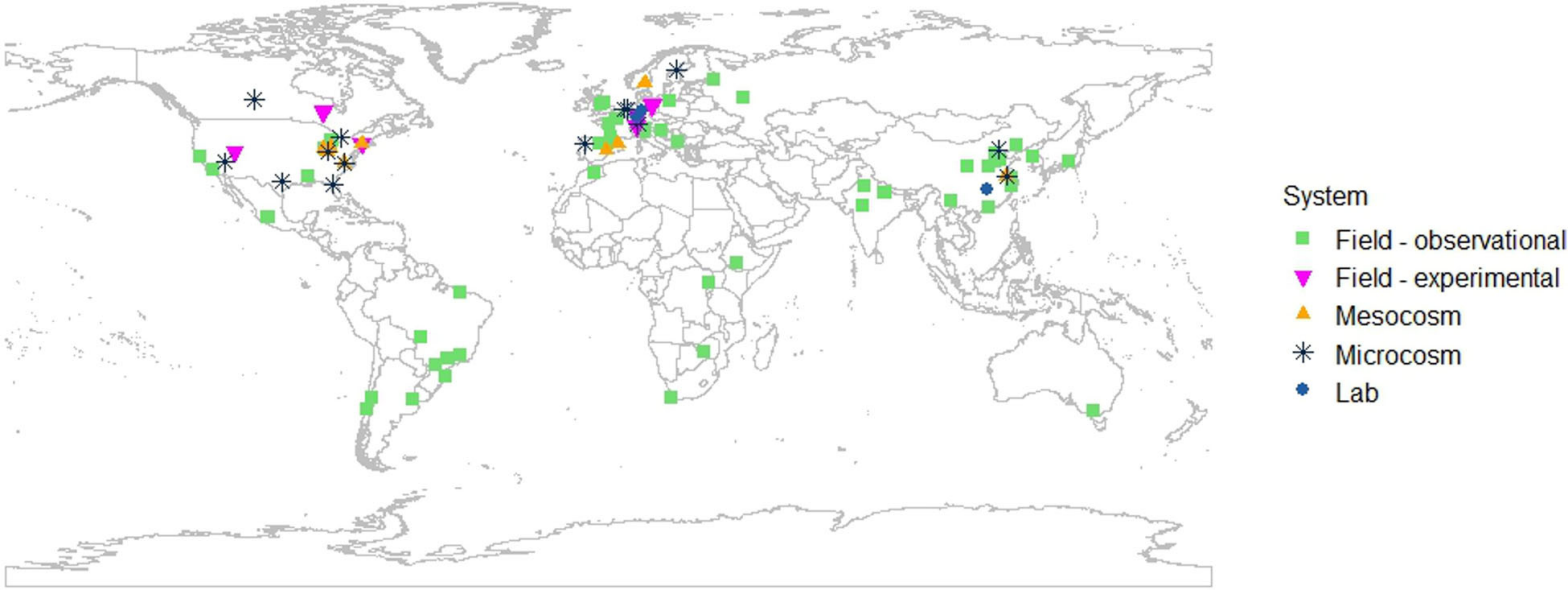
Geographical distribution of selected literature and study type: Six studies were experimental field studies, 51 were observational field studies, nine were outdoor mesocosms (semicontrolled bounded experimental ecosystems of ~>1 m^3^), 15 were indoor microcosms (some components and processes of natural ecosystems within bounded area of ~<1 m^3^), and three were laboratory studies (no creation of structures or processes of natural ecosystems). The microcosm and laboratory studies gave location information about where the study was carried out or where natural material used was collected. Five studies from the selected literature are not included because there was no location information

Studies measuring only aspects of aquatic ecosystem structure, such as species sensitivity, taxonomic biodiversity, richness, or taxonomic community structure, were excluded. Studies were also excluded if they examined a specific source of chemical stress, but only stated that the stressor had the potential to affect ecosystem function without quantifying any changes in a function. Likewise, descriptive studies comparing chemical or physical properties of different bodies of water were excluded if the text did not mention a down‐the‐drain chemical stressor or source of down‐the‐drain chemical stress. Reviews were also excluded. This application of selection criteria resulted in a database of 89 studies (Figure 1), with one or more measures that either directly or indirectly measured an ecosystem process (such as microbial respiration rate or net primary production), or that related to an attribute of a process (such as the biomass of the biofilm), or that measured the functional capacity of a process (such as the functional community composition of microbes; Table 1). We considered all these measures to be potential functional indicators. We did not include measures that were indicators of stressors, ecosystem structures, or ecosystem services (Box 1), although these measures were sometimes described as “functional” by the authors. Most of the selected literature was from the past three years, reflecting the recent rise in publications in this area (Figure S1). Studies done in North America, Europe, and China predominated, and nearly all the studies in tropical and subtropical freshwater systems were observational rather than experimental (Figure 2).

**Table 1:** The main measures categorized into four groups of potential functional indicators, with the number of references from the reviewed literature in parentheses

Functional indicator group	Potential functional indicators from the literature review	Indicates mainly	Measures
Rates of processes	Leaf litter breakdown rate (4)	Organic matter decomposition	Function
	Detritivore feeding rate (2)		
	Electron Transport System Activity of organic matter associated microbes (1)		
	Soil dehydrogenase activity (1)		
	Methane production rate (1)		
	Denitrification potential of biofilm (1)	Elemental cycling	
	Nitrogen dioxide flux (1)		
	Biochemical oxygen demand (15)	Metabolic functions	
	Microbial extracellular enzyme activity (5)		
	Amino acid uptake rate in biofilm (1)		
	Respiration rate (14)		
	(Ecosystem, community, biofilm, macrophytes, microbes)		
	Net or gross primary productivity (12)	Primary productivity	
	(From rate of oxygen production, carbon dioxide consumption, or rate of biomass production)		
	Photosynthesis rate of macrophytes (1)		
	Biomass production or growth rate (4) (invertebrates, zooplankton)	Secondary productivity	
States linked to processes	Biomass of fungi on leaves (1)	Organic matter decomposition	Structure
	Dissolved oxygen concentration (3)	Organic matter decomposition and metabolic functions	
	(used to calculate respiration)		
	Carbon measures (20)	Elemental cycling	
	Phosphorus measures (20)		
	Nitrogen measures (24)		
	Chlorophyll‐*a* concentration (17)	Primary productivity	
	Chlorophyll‐*b* concentration (1)		
	Biomass (16)		
	(Algae, biofilm, cyanobacteria, macrophytes)		
	Abundance (3) (diatoms, phytoplankton)		
	Volume (2) (algae)		
	Density (6) (algae, microbes in biofilm)		
	Cover of macrophytes (1)		
	Biomass (11) (microbes, invertebrates)	Secondary productivity	
	Abundance (7)		
	(Invertebrates, fish, microbes)		
	Density (2) (protozoa, prokaryotes)		
Status and composition of functional traits underlying processes	Detritivore feeding preferences (1)	Organic matter decomposition	Structure
	Sporulation of fungi on decomposing leaves (1)		
	Functional trait diversity/richness (4)	Multiple functions	
	(Invertebrates, microbes, phytoplankton)		
	Functional trait composition (20)		
	(Traits of diatoms, fish, invertebrates, macrophytes, microbial metabolic profile, phytoplankton)		
Processes measured at the food web level	Eco‐exergy (1)	Multiple and interacting functions at the food web level	Structure and function
	Niche uniformity of food web (1)		
	Dietary change of trophic groups (1)		
	Flow of energy through food web (1)		

**Figure 3:**
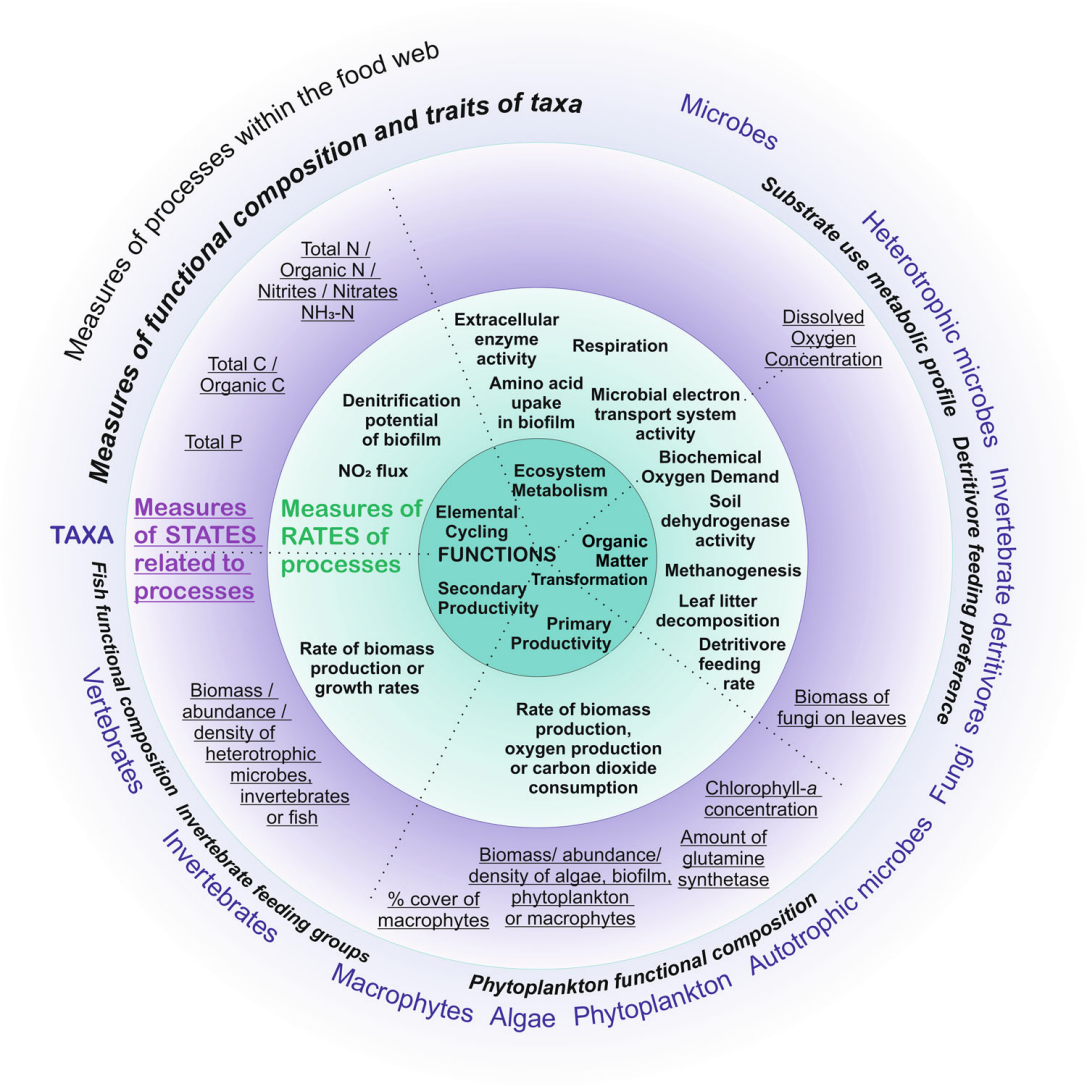
The conceptual relationships between potential indicators from the reviewed literature, the five ecosystem functions, and freshwater taxa. Some measures have been combined; further details are found in Table 1 and, for a full list of raw measures, see Supporting Information. The measures are categorized according to the five functions placed in the central dark green circle. The measures become less closely linked to ecosystem functions with movement from the inner to the outer concentric ring. The pale green inner ring contains measures that are rates of processes linked directly to a function. Moving outwards, the purple ring contains measures that are states of an aspect of a process at a fixed point in time. The blue outer ring shows the taxa frequently referred to in the literature arranged according to the functions they are most associated with. The measures of “functional composition and traits of taxa” are placed in this outer light blue ring because they describe the diversity, relative abundance, and/or composition of functional traits found within taxonomic groups, including microbes, fungi, and invertebrates that underpin multiple functions. Most of these measures of underpinning functional capacity contribute to multiple functions, so the dotted lines between the functions do not extend through the blue ring. The various food web metrics are also represented (although not listed) at the level of the blue ring because these measures describe processes and the relationships between taxa across trophic levels. Taxa are named as described in the literature, so the term “algae” is included in addition to phytoplankton and biofilm (periphyton). Biofilms include both autotrophic and heterotrophic microbes, so measures from many of the studies incorporate both primary productivity and secondary productivity

Although nutrients such as nitrogen and phosphorus can be considered as stressors themselves, their concentrations are also an attribute of nutrient cycling processes, so were also included as a potential functional indicator. For each of the 89 studies included in our review, we additionally recorded if any nonfunctional measures were assessed in these studies, to compare different types of indicators, and to examine how a variety of indicators might be used within ERA. We recorded the relevant taxon or trophic level of each measure. We then placed all the functional measures into categories according to how directly they measured processes contributing to one or more of five identified freshwater ecosystem functions (metabolic functions, nutrient cycling, organic matter transformation, primary productivity, and secondary productivity; Table 1; Figure 3). These five freshwater ecosystem functions were the most prominent in the literature (Burdon et al., 2020; Ferreira et al., 2020; Gessner & Chauvet, 2002; Peters et al., 2013; von Schiller et al., 2017; Young et al., 2008).

We then compared the methods and application of functional measures that occurred frequently in the reviewed literature against three good indicator criteria outlined by Jackson et al. (2000) and Kurtz et al. (2001): conceptual relevance, applicability, and response variability. Their other criteria about interpretation were designed for monitoring programs and were considered not relevant in an ERA context and hence were excluded from this analysis. We rated all selected functional measures using a point system against each of the three criteria to provide an overview of the most promising measures and the further development that would be required for their use as indicators within ERA (Table 2). When considering conceptual relevance, we rated measures as (1) (low relevance) measuring a capacity for a process or the result of a process indirectly linked to a function; (2) (medium) measuring a contributing process indirectly linked to a function; and (3) (high) measuring the main process that directly results in a function (Table 2). Please note that our rating of conceptual relevance is not identical to the categorization of types of measures shown in Figure 3 and Table 1. When considering applicability, we rated measures from 0 (low) to 3 (high) with a point given each for: clearly defined data collection methods, ease of implementation of these methods, and repeatability and robustness (Table 2). When considering response variability, we rated measures from 0 (unfavorable) to 3 (favorable) with a point given each for: low and well‐understood measurement error, low and well‐understood temporal variability, and low and well‐understood spatial variability (Table 2).

**Table 2:** The 13 most common measures of ecosystem function from literature included in our review, rated according to good ecological indicator criteria adapted from Jackson et al. (2000) and Kurtz et al. (2001) for relevance to ERA^a^

			Response variability
Potential indicators	Conceptual relevance 1 (low)–3 (high)	Applicability 0 (low)–3 (high)	0 (unfavorable)–3 (favorable)
Leaf litter breakdown rate	3	3	3
Net or gross primary productivity (rate of oxygen production, carbon dioxide consumption, or rate of biomass production)	3	3	3
Secondary productivity (biomass production or growth rate)	3	3	3
Respiration (ecosystem, community, microbial, biofilm, macrophytes)	3	2	3
Detritivore feeding rate	2	3	3
Chlorophyll‐*a* concentration	2	3	3
Biochemical oxygen demand	2	3	2
Extracellular enzyme activity	2	1	1
Invertebrate functional feeding groups	1	3	3
Nitrogen measures	1	3	1
Phosphorus measures	1	3	1
Organic carbon	1	3	1
Microbial metabolic profiles	1	2	2

*Note*: For further details see Table S2.

^a^ Conceptual Relevance asks: “Is the indicator relevant to the assessment question (management concern) and to the ecological resource or function at risk?” It is rated as one of the following: Measurement of the main process that directly results in a function (three points), Measurement of a contributing process indirectly linked to a function (two points) or Measurement of a capacity for a process, or the result of a process indirectly linked to a function (one point). Applicability asks: “Are the methods for sampling and measuring the indicator technically feasible, appropriate, and efficient for use in ecological risk assessment?” It is rated as 1 point each for: Methodological consistency—clearly defined data collection methods, Logistics—easy, cost‐efficient implementation and quality assurance—repeatable and robust. Response Variability asks: “Are errors of measurement and natural variability over time and space small and sufficiently understood and documented?” It is rated as 1 point each for: Measurement error—low and well understood, Temporal variability—low and well understood (within‐season and across‐year) and spatial variability—low and well understood.

## RESULTS

### Defining and categorizing potential functional indicators

Our review of published literature highlighted a lack of clarity in the use of the term ecosystem function, with some incorporating stressors (e.g., Johnson et al., 2019) or ecosystem services (e.g., Chen et al., 2019) into their definition. This creates potential confusion in the development of functional indicators for ERA. Measures that we considered potential indicators of function in freshwater ecosystems in response to down‐the‐drain chemical stress were rates or attributes of processes, or measures of functional effect traits (Box 1) underlying the capacity for these processes as summarized in Table 1. Measures that we considered unsuitable included direct measures of stressors, apart from those involved in elemental cycling, such as nitrogen and phosphorus, because these can be used to provide information about both the level of nutrient stress and processes involved in nutrient cycling. Other measures that were not considered potential indicators were measures of ecosystem structure, including species survival or sensitivity (such as damselfly larval survival or mortality of benthic macroinvertebrates) and taxonomic diversity and composition (such as community composition of macroinvertebrates or presence of invasive species). These decisions are based on the intrinsic definition of ecosystem function (Box 1) as the “net effect of processes that control fluxes of energy and matter through ecosystems, linking the various structural components of the ecosystem.” The last two columns of Table 1 highlight that there is a distinction between what is being measured and what is being indicated, because measures of some aspects of structure can sometimes be indicators of the capacity for processes and function (e.g., biomass as an indicator of production).

To shed light on the use of the term ecosystem function and related metrics, building on the analysis of the available literature, we identified four categories of potential functional indicators: process rates, functional states linked to processes, status and composition of functional traits underlying processes, and food web level process indicators (Table 1). We defined the first category, process rates, as aspects of particular functions measured as change over time, such as the rate of biomass generation (i.e., productivity). These processes vary in how directly linked they are to the function or how much they contribute to the function. The second category, states linked to processes, are measures of functional states at a fixed point in time that form part of a process, such as the amount of biomass. The third category, status and composition of functional traits underlying processes, represents the underlying capacity for processes and includes the relative abundance, diversity, presence, or attributes of particular traits, such as macroinvertebrate feeding preferences, phytoplankton functional composition, or microbial metabolic diversity. This is in contrast to taxonomic richness and measures of community composition that we considered to be structural. The fourth category, processes measured at the food web level, includes measures of the movement of biomass and energy through food webs. The metrics and methods used for food web analysis were unique to each study and generally not applicable to any one function, and so these measures have not been listed in full in Table 1 or in Figure 3.

Our classification approach highlights the different types of functional measures that could be used to provide evidence of ecosystem function during ERA. These four categories of potential functional indicators vary in how closely the aspects of the processes they measure are linked to one of the five ecosystem functions (such as nutrient cycling or production). The concentric rings in Figure 3 graphically represent the conceptual links between the categories of functional indicators and the relevant ecosystem functions (placed in the central dark green circle). Figure 3 also represents how these functional indicator categories are related to the taxa (placed in the outermost blue ring) that underpin the functions.

### Applicability and utility of potential functional indicators for ERA

The rating of these measures against good indicator criteria reveals high variability in how fit‐for‐purpose these measures currently are as indicators for use in ERA for down‐the‐drain chemicals (Table 2). The rate of leaf litter breakdown emerges as one of the measures with the highest readiness because it has (a) a high level of conceptual relevance, (b) high applicability, and (c) a reasonably well‐understood dose–response relationship (Tables 2 and S2). Various ways of measuring primary and secondary productivity also scored highly and could add important ecological context to ERA. Although measures such as detritivore feeding rates and chlorophyll‐*a* concentration are conceptually related, but effectively distant from a direct assessment of organic matter decomposition and primary productivity, these last ones might be evaluated by means of useful alternative measures. Biochemical oxygen demand and respiration could also provide useful information, although they could represent greater challenges with implementation and interpretation of change in relation to chemical stress.

The composition of invertebrate functional feeding groups is a commonly used measure, but it should be used alongside other measures more closely linked to functions to fully understand chemical stress impacts. Various measures of nutrient status are relatively straightforward to implement, but their use in ERA may be limited because repeated sampling and careful interpretation would be needed to understand chemical stress impact on the various functional processes involved in nutrient cycling (Table S2). In contrast, although considerable further methodological development is needed for the application of extracellular enzyme activity and microbial metabolic profiles within ERA, these have great potential to provide currently missing information about microbial metabolic function (Table 2).

## DISCUSSION

### Implications for definitions of ecosystem function

There is the potential for confusion in the various uses of the term ecosystem function in the wider literature, with inconsistent terminology around ecosystem structures, processes, and functions (Cadotte et al., 2011; de Groot et al., 2002; Farnsworth et al., 2017; Jax, 2005; Odum, 1962). This lack of clarity is likely to hinder the consideration and development of functional indicators within ERA. Importantly, we identified that there is frequently some blurring of the boundaries between structure and function (de Groot et al., 2002; Spangenberg et al., 2014). For example, measures of biomass might initially appear to be indicators of structure, but are also directly related to productivity and thus are useful as functional indicators. Given that the same biomass could be provided by different biological components (e.g., species), all other factors remaining equal, a measurement of the rate of biomass accumulation could be considered a functional indicator of ecosystem productivity. In a similar way, the state of a macroinvertebrate trait might appear to be measuring ecosystem structure (e.g., feeding preference), but it is indicating the capacity for delivering one or more functions (such as organic matter transformation). Therefore, it is helpful to distinguish between what is being measured and the ecosystem function or structural change that is being indicated by these measures, as we have done in our analysis.

In addition to a lack of clarity around terminology related to structure and function, some authors combine definitions of processes with function (e.g., Jax, 2005; Spangenberg et al., 2014). For ERA, this may lead to the inherent difficulty of assuming that a single indicator directly measures a change in function, rather than being a measure of a process contributing to a function that can be used as an indicator. This is particularly evident in the distinction between measuring processes versus measuring the underlying capacities for processes. A functional effect trait (Box 1) represents the underlying capacity to influence processes that contribute to function, and so change in the prevalence of a trait does not necessarily translate directly into changes in the ecosystem function itself (Díaz et al., 2013; Farnsworth et al., 2017). Much of the literature on incorporating greater ecological relevance and functional indicators into ERA has concentrated on functional traits and functional groups (Segner, 2011; Van den Berg et al., 2019; Van den Brink et al., 2011), but including indicators of functional processes places greater emphasis on the underlying ecological mechanisms embedded within ecosystems and is likely to provide more direct information on potential impacts of stress. We argue that the term ecosystem function should be used to represent the combined effect of multiple processes relevant to a particular function, such as organic matter transformation or metabolic functions. Indicators of this function could include a direct measure of one of these processes, an indirect measure of an aspect of a process, or a measure of a capacity for these processes. In contrast, “ecosystem functioning” combines multiple functions and carries with it an implied judgment about maintaining ecosystems in a safe operating space.

### Different types of functional indicator

We demonstrate that there is considerable variation in types of functional measure, both conceptually and in their practical implementation. Functional measures represent different attributes of processes that make up a particular function. Processes and measures are highly interconnected and frequently “nested” within each other. For example, the biomass of fungi on leaves, and the functional composition and feeding preferences of macroinvertebrate communities are all connected to the rate of leaf litter decomposition. This means that related measures can be used in combination to gain information about a function within ERA.

Different ecosystem functions are also highly linked. For example, processes involved in elemental cycling are also important for organic matter transformation. In contrast to conventional ERA approaches that usually focus on individual populations, our analysis demonstrates that functional indicators span different levels of biological organization, including metabolic gene diversity and enzyme activity in biofilms, through to metrics of the whole food web. This highlights the benefits of including functional indicators in ERA for down‐the‐drain chemicals to account for impacts that may happen across the trophic chain and indirect effects. Metrics of food web dynamics often incorporate multiple structural and functional measures and are useful for revealing change in the flow of energy within the whole food web that affects multiple functions (Price et al., 2019). The potential functional indicators we have identified in this review also operate at different trophic levels, such as primary producers, decomposers, and consumers.

The classification presented here highlights that potential indicators vary in how directly they measure processes or the underlying capacities for processes to occur. The four described categories of potential functional indicators encompass the full range of studies we reviewed and represent the different ways in which processes can be measured and used to indicate change in function on down‐the‐drain chemical stress. This classification provides a range of options for assessing change in ecosystem functions and aids understanding of how different aspects of processes contribute to a particular function and to overall ecosystem functioning, rather than there being one simple indicator of function.

### Identifying the most useful potential functional indicators for ERA

Some measures reveal potential for development within ERA applications to down‐the‐drain chemicals. The rate of leaf litter breakdown in particular provides information about a key process that integrates other ecosystem structures and functions and demonstrates good sensitivity to chemical stress (Chauvet et al., 2016; Ferreira et al., 2020; Gessner & Chauvet, 2002; Lemes da Silva et al., 2020; Young et al., 2008). However, it will be important to distinguish between the use of this measure as an indicator in bioassessment programs, where the integrity of a particular ecosystem under multiple stressors is the focus, and its use within an ERA context, where the focus is on predicting impacts of particular chemical stressors (Ferreira et al., 2020; Xu et al., 1999). This translation of measures from field‐based biomonitoring to ERA approaches will be particularly important in the development of measures that add information on the impacts on and the role of the microbial community. Although extracellular enzyme activity and microbial metabolic substrate use profiles were rated the lowest, they have strong potential for further development for ERA by translating understanding of metabolic processes and microbial activity in relation to particular chemical stressors between laboratory‐based, microcosm, mesocosm, and field studies (Burdon et al., 2020; Miao, Guo, et al., 2019). However, distinguishing the response of such profiles to chemical stress compared with other stressors and temporal and spatial variability remains challenging (e.g., Millar et al., 2015).

Other measures that were identified, such as biochemical oxygen demand and rate of biomass production, provide valuable information about organic matter transformation and respiration, but these will need to be interpreted carefully in relation to the wider ecosystem context and may be challenging to use for the assessment of specific chemical stress within an ERA framework. There is a lack of consistent data with which to evaluate relative sensitivity and specificity among these different types of potential functional indicators. Sensitivity may vary with the type of chemical stress and ecosystem context, rather than being an inherent property of a particular type of indicator. Moreover, any changes detected by different indicators could be caused by other factors, not necessarily by chemical stress. Evidence about chemical stressor impacts will require developing multiple lines of evidence assessing change in various processes contributing to ecosystem function.

### Using a range of indicators for increased sensitivity of ERA

It is clear that one type of indicator can be sensitive to chemical stress that is not detected by another type of indicator; thus functional indicators add value to traditional ERA approaches by detecting sublethal impacts of chemical stressors that may not be detected by indicators based on species composition or species‐level toxicity data. Peters et al. (2013) used a quantitative review of 122 studies to investigate the relationship between risk assessed using standard single‐species toxicity test data (i.e., *Daphnia*, algae) and toxicant effects on ecosystem functions (i.e., leaf litter breakdown, primary production, and community respiration). Although they found some evidence of apparent effects on ecosystem functions at concentrations below regulatory thresholds (i.e., toxic units <0.01), there was no relationship between toxic units and ecosystem function effects. Sandin and Solimini (2009) provide examples of detecting change in function without detecting change in structure for a variety of stressors, supporting the inclusion of a range of different indicators in ERA.

It is also possible to detect change in structural indicators and indicators of functional composition, without detecting direct change in a functional process. A study in our review demonstrated that taxonomic and functional diversity indicators were reduced in response to high concentrations of nanoplastics, without any change in an indicator of functional process (the ability to metabolize carbon substrates; Miao, Guo, et al., 2019). Impacts on a function might first be detected by a direct change in a rate of an ecosystem process (such as rate of leaf litter breakdown), by a change in an attribute of that process (such as biomass of macroinvertebrate leaf shredders), by a change in the abundance of functional traits underlying processes (such as functional composition of macroinvertebrates), or by a change in the whole food web structure.

Different functions can also report different impacts of a chemical stressor. For instance, measures of rates of microbial metabolic processes (e.g., enzyme activity) and microbial metabolic functional composition (e.g., capacity to metabolize substrates) can be affected by a chemical stressor, without change in measures of productivity (Perujo et al., 2016). Perujo et al. examined impacts of effluent from wastewater treatment plants on river sediment biofilm communities, and relying on productivity‐related measurements alone would not have revealed the detrimental impacts on biofilm metabolic responses. Further development and a careful interpretation of multiple measures will be needed within ERA to build a picture about possible change in ecosystem functions in response to chemical stress, rather than assuming that any one measure straightforwardly indicates a particular function or the maintenance of safe operating space for an ecosystem.

Our study focused on the importance of functional indicators, but it nonetheless remains important to consider impacts on individual species (Belanger et al., 2000). This highlights the inherent difficulty in prioritizing single‐species testing within ERA over considering functional endpoints (and vice versa) and the need to assess a range of indicators on a case‐by‐case basis to capture the complex ecosystem responses to chemical stressors. We suggest that a valuable framework for incorporating functional indicators into ERA will be to choose a rate and/or a state measure of a process and a related “capacity for process” indicator (Figure 3), alongside one or more structural indicators of the sensitivity or diversity of relevant taxa. Where monitoring is possible over time, multiple measures could be combined to provide the information needed to study system level responses such as change in biomass and energy movement through the food web (e.g., the use of exergy by Xu et al., 1999; Zhang et al., 2013). This combined approach is most likely to provide both greater sensitivity to impacts and greater ecological relevance; it promises to build evidence about the mechanisms underpinning the responses to impacts and to disclose functional compensation when it may be occurring (Box 1).

## FUTURE DIRECTIONS

### Further information required to build the evidence base for functional indicators in ERA

The potential functional indicators we have highlighted from our review are not yet ready to be incorporated into conventional ERA due to a lack of data on their sensitivity and specificity to down‐the‐drain chemical stress. This lack of information highlights fundamental issues about ecological context that are embedded in the concept of ecosystem function. Data from functional indicators cannot be incorporated into existing ERA frameworks in the same way that toxicity data can be used with structural endpoints, because ecosystem context, indirect effects, and multiple stressors can all influence how stressors affect function. Thus, data collection and modeling of the responses of potential functional indicators to stressors need to incorporate ecological context (Segner, 2011; Stark, 2005; Van den Brink, 2008; Woodward et al., 2012). Mesocosm experiments provide an opportunity to investigate real‐world scenarios by blending multifactorial experimental stresses within more ecologically relevant laboratory settings (Baho et al., 2019; Belanger et al., 2000; Perujo et al., 2016), although they are frequently resource‐intensive.

Many of the studies in our review addressed wastewater with a combination of nutrient enrichment and down‐the‐drain chemicals and the potential for complex interactions (Birk et al., 2020; Côté et al., 2016; Hering et al., 2015). Modeling approaches may be useful for exploring the impact of multiple stressors on particular processes (Galic et al., 2017). Although it may be technically feasible to extrapolate from laboratory to ecosystem conditions, and from individual effects to population and ecosystem‐level effects, obtaining data to calibrate ecosystem‐level models is a major challenge (Franco et al., 2017). Franco et al. (2017) suggest focusing on vulnerable species and traits, but our review highlights the value of including the additional consideration of functional indicators that are more directly connected with mechanistic processes. However, there is a need to understand the extent to which the use and interpretation of different functional indicators are transferable between chemical stressors, different sites, and between laboratory and field contexts to reveal impacts, compensation, and recovery.

Our review has highlighted the importance of considering the effects of down‐the‐drain stressors on structure and functions across multiple levels of organization (genes, individuals, populations, communities, and ecosystems; Fleeger et al., 2003) and across different trophic levels. There is a particular need to develop reliable indicators for processes and the capacities for processes driven by microbial communities, which are currently poorly considered within ERA (Arias‐Andres et al., 2018; Blunt et al., 2018; Burdon et al., 2020; Miao, Guo, et al., 2019; Miao, Wang, et al., 2019; Rosi‐Marshall & Royer, 2012). Changes in microbial substrate use profiles over time using Biolog MicroPlates might be a robust way to assess rapid change in metabolic function for ERA (Blunt et al., 2018; Oest et al., 2018), but further evidence is needed to assess the sensitivity and specificity of direct and indirect effects in response to different stressors.

## CONCLUSION

Reaching a consensus on definitions of ecosystem function and the different measurements that contribute to a function will provide greater clarity for ERA. We have clarified the distinction between different types of functional indicators, such as between measures of processes and measures of capacities for processes. This distinction has highlighted the importance of considering process‐based measures as functional indicators, rather than only focusing on measuring functional traits. Our review highlights that understanding and incorporating different types of functional indicators into ERA would improve the sensitivity of the approach and provide greater ecosystem relevance and a holistic consideration of ecosystem vulnerability (Segner, 2011). Such a change in emphasis will have wider implications for understanding and mitigating the impacts of multiple stressors (including nutrients), helping to improve the resilience of aquatic ecosystems, and providing information for ecosystem restoration (Angeler et al., 2014; Beechie et al., 2010; Brock et al., 2018) and the consideration of impacts on ecosystem services (Bruins et al., 2017).

## CONFLICT OF INTEREST

The authors declare that there are no conflicts of interest.

## DISCLAIMER

The peer review for this article was managed by the Editorial Board without the involvement of C. Rivetti.

## Supporting information

This article contains online‐only Supporting Information.

A list of selected literature.
A figure of the number of publications obtained in the intial literature search and that were selected for inclusion in this review, organized by year of publication.

A figure of measures of process rates and states arranged by ecosystem function, as described in Figure 3.

Supporting Information

A spreadsheet showing all functional measures from the selected literature and how these were categorized and summarized.

A table giving further detail about the scores given to 13 potential indicators based on good indicator criteria in Table 2.

## Data Availability

The list of reviewed literature and of reviewed measures are available as supplemental files.
