# Supplementary material for: Functional measures as potential indicators of down‐the‐drain chemical stress in freshwater ecological risk assessment
Source: Integr Environ Assess Manag. 2022 Jan 18;18(5):1135–47. doi: 10.1002/ieam.4568 (PMC9543243; doi:10.1002/ieam.4568)
Supplement: Supplementary file 1 — A list of selected literature. A figure of the number of publications obtained in the intial literature search and that were selected for inclusion in this review, organized by year of publication. [file IEAM-18-1135-s004.docx]

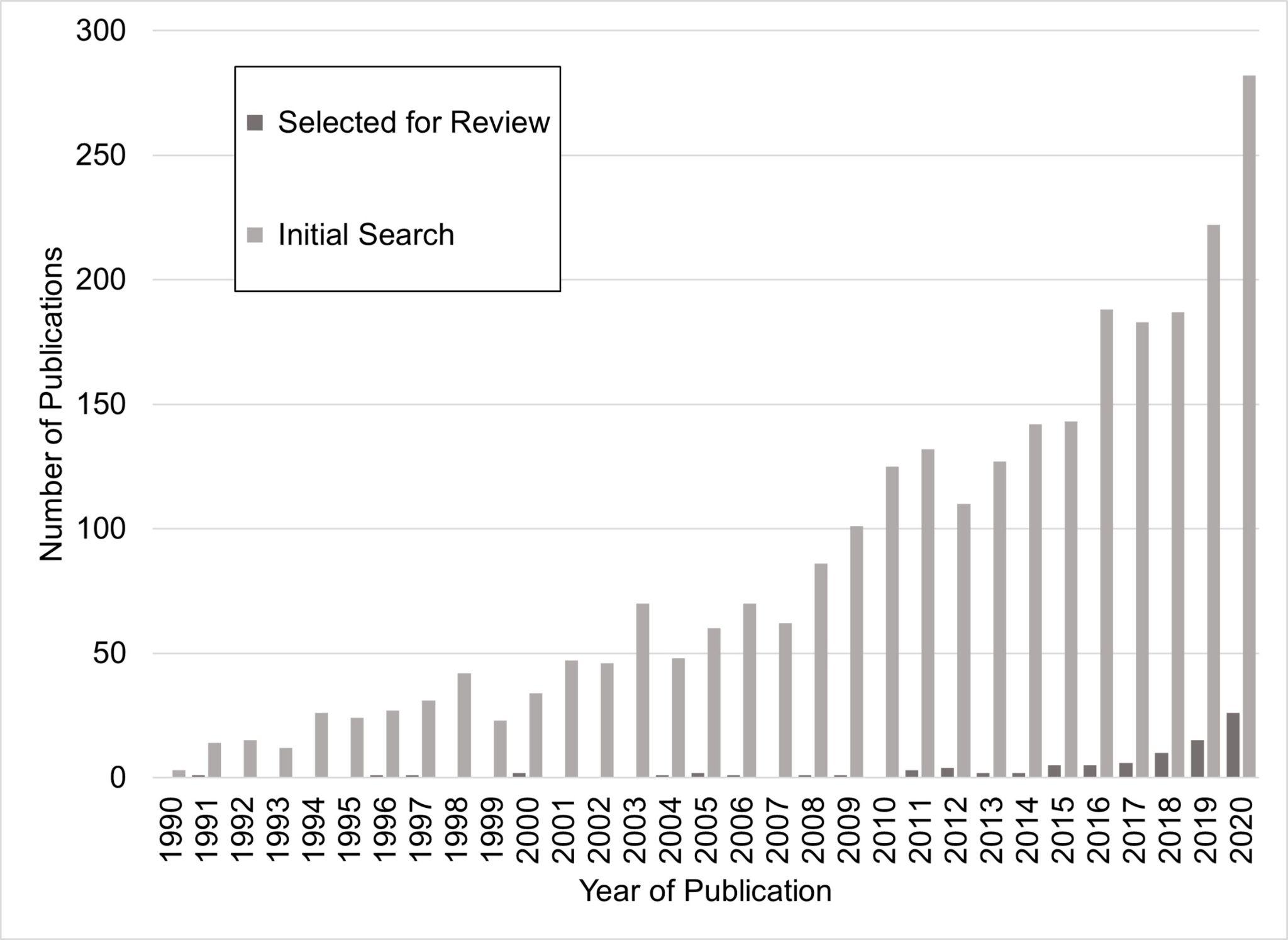


Supplemental Figure 1: The number of publications that were obtained in the initial literature search (2688 papers) and that were selected for inclusion in this review (89 papers), organised by year of publication.
