# Supplementary material for: Functional measures as potential indicators of down‐the‐drain chemical stress in freshwater ecological risk assessment
Source: Integr Environ Assess Manag. 2022 Jan 18;18(5):1135–47. doi: 10.1002/ieam.4568 (PMC9543243; doi:10.1002/ieam.4568)
Supplement: Supplementary file 2 — A figure of measures of process rates and states arranged by ecosystem function, as described in Figure 3. [file IEAM-18-1135-s002.docx]

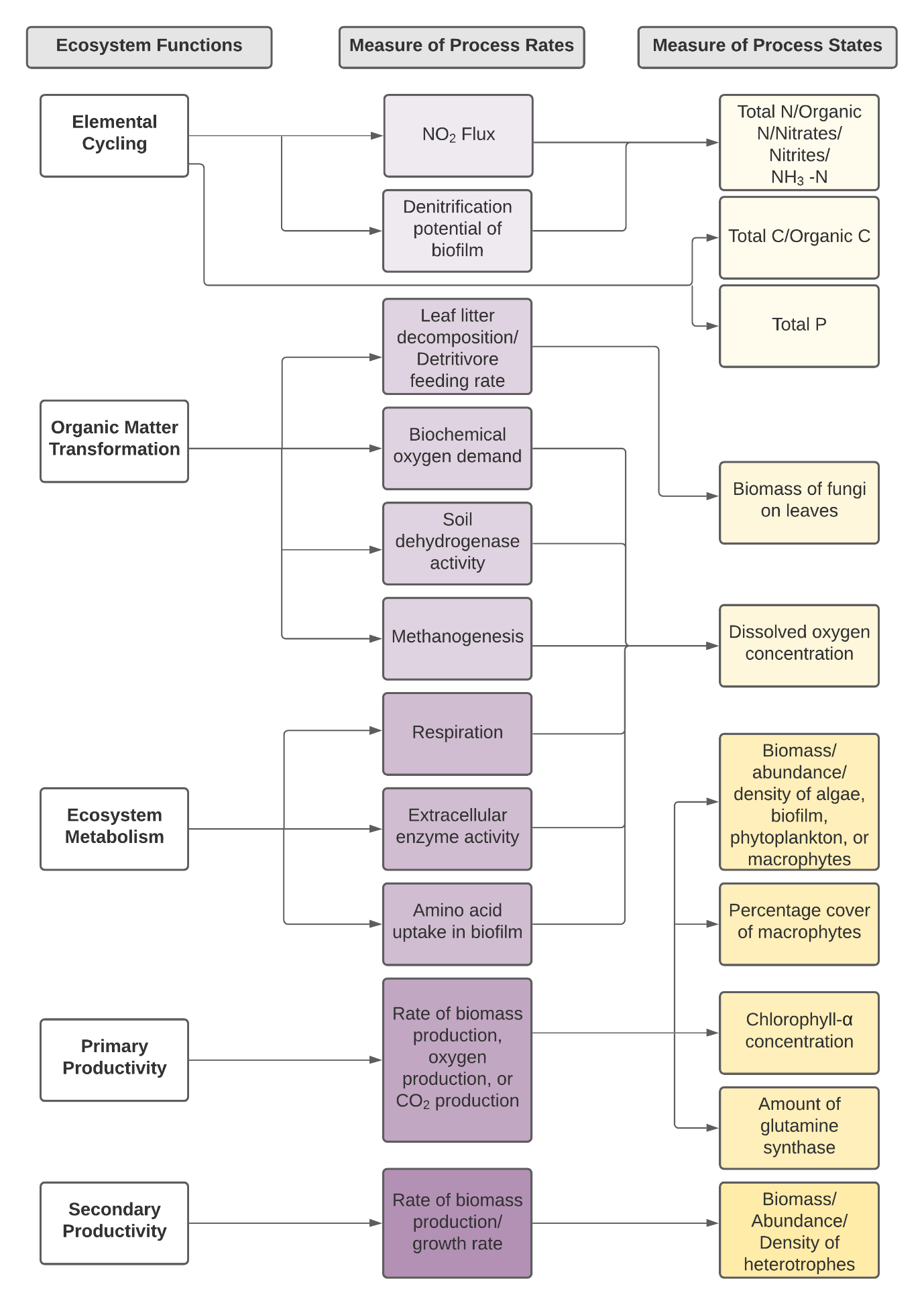


Supplementary Figure 2. Measures of process rates and states arranged by ecosystem function, as described in Figure 3.
