# Supplementary material for: Functional measures as potential indicators of down‐the‐drain chemical stress in freshwater ecological risk assessment
Source: Integr Environ Assess Manag. 2022 Jan 18;18(5):1135–47. doi: 10.1002/ieam.4568 (PMC9543243; doi:10.1002/ieam.4568)
Supplement: Supplementary file 5 — A table giving further detail about the scores given to 13 potential indicators based on good indicator criteria in Table 2. [file IEAM-18-1135-s005.docx]

**Supplemental to Table 2.** Explanation of the scores given to 13 potential indicators based on good indicator criteria in Table 2, according to good ecological indicator criteria adapted from Kurtz and colleagues for relevance to Ecological Risk Assessment [(Jackson et al. 2000; Kurtz et al. 2001)](https://paperpile.com/c/eawJdl/EOe1+JJmU): i) Conceptual Relevance ii) Applicability, iii) Response Variability.

|  |  | **Good Indicator Criteria** |  |
| --- | --- | --- | --- |
|  | **Conceptual Relevance** | **Applicability** | **Response Variability** |
| **Questions addressed by criteria:** | **Is the indicator relevant to the assessment question (management concern) and to the ecological resource or function at risk?** | **Are the methods for sampling and measuring the environmental variables technically feasible, appropriate, and efficient for use in a monitoring program?** | **Are errors of measurement and natural variability over time and space sufficiently understood and documented?** |
| **Ratings of Criteria:**  **(Maximum of 3 points)** | **One of the following:**   - **Measurement of the main process that results in a function (3 points)** - **Measurement of a contributing (and nested) process linked to a function (2 points)** - **Measurement of a capacity for a process, or a state resulting from a process linked to a function (1 point)** | **One point each for:**   - **Methodological consistency - clearly defined data collection methods** - **Logistics - easy, cost-efficient implementation** - **Quality assurance - repeatable and robust** | **One point each for:**   - **Measurement error - low and well understood** - **Temporal variability - low and well understood (within-season & across-year)** - **Spatial variability - low and well understood** |
| **Potential Indicators** |  |  |  |
| **Leaf litter breakdown rate** | 3: Measuring the main process that results in decomposition. | 3: Methods are well-defined, easy to carry out, and methods are repeatable. The methods are not costly relative to other methods. Data collection methods commonly include using mesh bags containing leaf litter, placed in the water at a site, and change in leaf litter mass measured through weighing ash free dry leaf mass [(Bundschuh et al. 2011; Jonsson et al. 2015; Lemes da Silva et al. 2020; Lin et al. 2020)](https://paperpile.com/c/eawJdl/ykT7+XEh2+rmsd+42Yy) or loss in mass of leaf discs following freeze-drying [(Pimentão et al. 2020)](https://paperpile.com/c/eawJdl/lwMp). Rate of breakdown is calculated using change in mass over time. | 3: The effects of temporal and spatial variability are well understood due to variations in leaf litter breakdown across and within seasons being well-researched. Measurement error is also well understood for the leaf litter bag method due to its common usage. |
| **Primary Productivity (rate of oxygen production, carbon dioxide consumption or rate of biomass production)** | 3: Measuring the main process that results in primary productivity. | 3: Methods are well-defined and logistically simple to implement, as well as being repeatable. Measured via oxygen analysis of water samples from the field - the bottle incubation method [(e.g. Shirokova et al. 2016)](https://paperpile.com/c/eawJdl/4nJk/?prefix=e.g.). | 3: The effects of temporal and spatial variability on rates of primary productivity are well understood in the associated literature. Measurement error is also well understood. |
| **Secondary Productivity (rate of biomass production or growth)** | 3: Measuring the main process that results in secondary productivity. | 3: Methods are well-defined and logistically simple to implement, as well as being repeatable. Repeated measures such as body length, head capsule length or weight over time [(Wilson et al. 2004; Redondo-Hasselerharm et al. 2018; Kidd et al. 2020)](https://paperpile.com/c/eawJdl/pi4z+w4Io+YKvH). Weight might be estimated from lengths and Instantaneous Production calculated from weights, water temperature and existing models for that taxon [(Aguiar et al. 2015)](https://paperpile.com/c/eawJdl/OnDy). | 3: The effects of temporal and spatial variability on secondary productivity are well understood in the associated literature. Measurement error is also well understood. |
| **Respiration (ecosystem, community, microbial, biofilm, macrophytes)** | 3: Measuring the main process that results in respiration. | 2: Methods are well-defined and easy to carry out, however less repeatable and robust at an ecosystem and community scale. The methods are not costly relative to other methods. Respiration was measured via continuous DO measurements daily or monthly using a EXO1 data sondes probe (YSI) [(Simonin et al. 2018)](https://paperpile.com/c/eawJdl/4xHk). | 3: The effects of temporal and spatial variability on respiration are well understood. Measurement error is also well understood due to methods of measurement being well defined. |
| **Detritivore feeding rate** | 2: Measuring a contributing (and nested) process linked to the decomposition function, as detritivores are partly responsible for leaf litter breakdown through their feeding rate on leaves. | 3: Feeding rate is measured by consumed leaf mass per animal per day [(Bundschuh et al. 2011; Redondo-Hasselerharm et al. 2018)](https://paperpile.com/c/eawJdl/42Yy+pi4z) this requires collection and identification of detritivores, sediments and leaf litter inside of beakers, followed by weighing the leaf mass at start and end of the study, and incorporating change in species numbers by the end of the study. Methods are clearly defined and logistically simple to carry out, as well as being repeatable. | 3: The effects of temporal and spatial variability on the rate of detritivore feeding is well studied and understood. Measurement error is also well understood due to having well-used methods. |
| **Chlorophyll a** | 2: Measuring a contributing (and nested) process linked to the ecosystem function (primary productivity) as chlorophyll a is a crucial component in photosynthesis. | 3: Measured by spectrophotometry of water samples from the field (µg/L) [(Shirokova et al. 2016)](https://paperpile.com/c/eawJdl/4nJk) and also using fluorescence detection [(Wilson et al. 2004; He et al. 2019)](https://paperpile.com/c/eawJdl/djJs+YKvH). Methods are predefined, easy and cost-effective to carry out, and are repeatable. | 3: The effects of temporal and spatial variability of chlorophyll*-a* concentrations are well understood. Measurement error is also well understood for methods commonly used to measure chlorophyll a. |
| **Biochemical Oxygen Demand** | 2: Measuring a contributing (and nested) process linked to metabolism rate, or organic matter transformation. | 3: Biochemical oxygen demand has been measured in the reviewed literature by measuring oxygen before and after a 5-day incubation period (BOD_5_) [(Siddiqui and Pandey 2019; Donoso and Rios-Touma 2020; Fouzi et al. 2020; Medupin et al. 2020; Musonge et al. 2020)](https://paperpile.com/c/eawJdl/rC4p+OkYa+4ry3+4WDp+8rzK) using standard methods as in Rice et al [(2017)](https://paperpile.com/c/eawJdl/2DF7/?noauthor=1). However, there are modifications and alternative ‘second generation’ methods that decrease variability and analysis time [(Jouanneau et al. 2014)](https://paperpile.com/c/eawJdl/Zn37). Methods are predefined, easy to carry out, and methods are repeatable. The methods are not costly relative to other methods. | 2: Estimation of measurement error is not well understood when specifically using in ERA, as biochemical oxygen demand fluctuates with chemical stress and species present. Temporal and spatial variability are well understood. |
| **Extracellular Enzyme Activity** | 2: Measuring a contributing (and nested) process linked to ecosystem metabolism. | 1: Millar et al [(2015)](https://paperpile.com/c/eawJdl/z9Rg/?noauthor=1) used high throughput microplate assays to measure extracellular enzyme activity. Methods are not well defined for all extracellular enzymes, making them less easy to carry out and difficult to repeat. The methods are not costly relative to other methods. | 1: The effects of temporal and spatial variability are not well understood due to the large variation in enzymes that can be focused on. Measurement error is well understood. |
| **Invertebrate Functional Feeding groups** | 1: Measurement of a capacity for a process, or a state resulting from a process linked to secondary productivity. | 3: Functional feeding groups are ascertained by sorting, counting, and identifying the samples according to taxonomic references [(Wang et al. 2021)](https://paperpile.com/c/eawJdl/3E9f). Methods are predefined, easy to carry out, and methods are repeatable. The methods are not costly relative to other methods. | 3: The effects of temporal and spatial variability are well understood as invertebrate behaviour and life cycles have been well-studied. Measurement error is also well understood as this method is commonly used. |
| **Nitrogen measures** | 1: Measurement of a capacity for a process, or a state resulting from a process linked to the elemental cycling function. | 3: Nitrogen was measured on a Shimadzu TOC‐L instrument by high‐temperature catalytic oxidation by He et al [(2019)](https://paperpile.com/c/eawJdl/djJs/?noauthor=1). Methods are predefined, easy to carry out, and methods are repeatable. The methods are cost-effective. | 1: Estimation of measurement error is well understood, but temporal and spatial variability is less well understood due to fluctuations in levels of nitrogen being caused by a multitude of factors. |
| **Phosphorous Measures** | 1: Measurement of a capacity for a process, or a state resulting from a process linked to the elemental cycling function. | 3: Phosphorus was measured by UV‐Vis spectroscopy (Thermo Scientific) by He et al [(2019)](https://paperpile.com/c/eawJdl/djJs/?noauthor=1). Methods are predefined, easy to carry out, and methods are repeatable. The methods are cost-effective. | 1: Estimation of measurement error is well understood, but temporal and spatial variability of phosphorus in situ is less well understood, as it can fluctuate a great deal. |
| **Organic Carbon** | 1: Measurement of a capacity for a process, or a state resulting from a process linked to the elemental cycling function. | 3: Organic carbon was measured on a Shimadzu TOC‐L instrument by high‐temperature catalytic oxidation by He et al [(2019)](https://paperpile.com/c/eawJdl/djJs/?noauthor=1). Methods are predefined, easy to carry out, and methods are repeatable. The methods are cost-effective. | 1: Estimation of measurement error is well understood, but temporal and spatial variability is less well understood due to a variety of factors affecting organic carbon levels in ecosystems. |
| **Microbial metabolic profiles** | 1: Measurement of a capacity for a process, or a state resulting from a process linked to various ecosystem functions e.g. leaf litter breakdown, metabolism, nutrient cycling. | 2: The Biolog EcoPlate method was used by Miao et al [(2019)](https://paperpile.com/c/eawJdl/KveQ/?noauthor=1) and Blunt et al [(2018)](https://paperpile.com/c/eawJdl/e76S/?noauthor=1). Methods are predefined and easy to carry out. However, it is difficult to replicate exact results upon repeating methods due to high variability of microbial communities. The methods are not costly relative to other methods. | 2: Measurement error and temporal variability is well understood, but spatial variability of microbial communities is less well understood at different scales. |

Literature cited in Supplemental Table 2

[Aguiar ACF, Gücker B, Brauns M, Hille S, Boëchat IG. 2015. Benthic invertebrate density, biomass, and instantaneous secondary production along a fifth-order human-impacted tropical river. Environ Sci Pollut Res Int. 22(13):9864–9876.](http://paperpile.com/b/eawJdl/OnDy)

[Blunt SM, Sackett JD, Rosen MR, Benotti MJ, Trenholm RA, Vanderford BJ, Hedlund BP, Moser DP. 2018. Association between degradation of pharmaceuticals and endocrine-disrupting compounds and microbial communities along a treated wastewater effluent gradient in Lake Mead. Sci Total Environ. 622-623:1640–1648.](http://paperpile.com/b/eawJdl/e76S)

[Bundschuh M, Pierstorf R, Schreiber WH, Schulz R. 2011. Positive effects of wastewater ozonation displayed by in situ bioassays in the receiving stream. Environ Sci Technol. 45(8):3774–3780.](http://paperpile.com/b/eawJdl/42Yy)

[Donoso JM, Rios-Touma B. 2020. Microplastics in tropical Andean rivers: A perspective from a highly populated Ecuadorian basin without wastewater treatment. Heliyon. 6(7):e04302.](http://paperpile.com/b/eawJdl/rC4p)

[Fouzi TA, Youness M, Bouchra L, Ali B. 2020. Spatio-temporal typology of the physico-chemical parameters of a large North African river: the Moulouya and its main tributaries (Morocco). Afr J Aquat Sci. 45(4):431–441.](http://paperpile.com/b/eawJdl/4WDp)

[He D, He C, Li P, Zhang X, Shi Q, Sun Y. 2019. Optical and Molecular Signatures of Dissolved Organic Matter Reflect Anthropogenic Influence in a Coastal River, Northeast China. J Environ Qual. 48(3):603–613.](http://paperpile.com/b/eawJdl/djJs)

[Jackson LE, Kurtz JS, Fisher WS. 2000. Evaluation guidelines for ecological indicators. EPA/620/R-99/005. U.S. Environmental Protection Agency, Office of Research and Development, Research Triangle Park, NC.](http://paperpile.com/b/eawJdl/EOe1) <https://archive.epa.gov/emap/archive-emap/web/pdf/ecol_ind.pdf>[.](http://paperpile.com/b/eawJdl/EOe1)

[Jonsson M, Ershammar E, Fick J, Brodin T, Klaminder J. 2015. Effects of an antihistamine on carbon and nutrient recycling in streams. Sci Total Environ. 538:240–245.](http://paperpile.com/b/eawJdl/XEh2)

[Jouanneau S, Recoules L, Durand MJ, Boukabache A, Picot V, Primault Y, Lakel A, Sengelin M, Barillon B, Thouand G. 2014. Methods for assessing biochemical oxygen demand (BOD): a review. Water Res. 49:62–82.](http://paperpile.com/b/eawJdl/Zn37)

[Kidd KA, Graves SD, McKee GI, Dyszy K, Podemski CL. 2020. Effects of Whole-Lake Additions of Ethynylestradiol on Leech Populations. Environ Toxicol Chem. 39(8):1608–1619.](http://paperpile.com/b/eawJdl/w4Io)

[Kurtz JC, Jackson LE, Fisher WS. 2001. Strategies for evaluating indicators based on guidelines from the Environmental Protection Agency’s Office of Research and Development. Ecol Indic. 1(1):49–60.](http://paperpile.com/b/eawJdl/JJmU)

[Lemes da Silva AL, Lemes WP, Andriotti J, Petrucio MM, Feio MJ. 2020. Recent land-use changes affect stream ecosystem processes in a subtropical island in Brazil. Austral Ecol. 45(5):644–658.](http://paperpile.com/b/eawJdl/rmsd)

[Lin Q, Zhang Y, Marrs R, Sekar R, Luo X, Wu N. 2020. Evaluating ecosystem functioning following river restoration: the role of hydromorphology, bacteria, and macroinvertebrates. Sci Total Environ. 743:140583.](http://paperpile.com/b/eawJdl/ykT7)

[Medupin C, Bannister C, Schwartz J-M. 2020. Exploring the Interactions of Physical, Chemical and Biological Variables of an Urban River Using Network Analysis. Water. 12(9):2578.](http://paperpile.com/b/eawJdl/OkYa)

[Miao L, Guo S, Liu Z, Liu S, You G, Qu H, Hou J. 2019. Effects of nanoplastics on freshwater biofilm microbial metabolic functions as determined by BIOLOG ECO microplates. Int J Environ Res Public Health. 16(23). doi:](http://paperpile.com/b/eawJdl/KveQ)[10.3390/ijerph16234639](http://dx.doi.org/10.3390/ijerph16234639)[.](http://paperpile.com/b/eawJdl/KveQ) <http://dx.doi.org/10.3390/ijerph16234639>[.](http://paperpile.com/b/eawJdl/KveQ)

[Millar JJ, Payne JT, Ochs CA, Jackson CR. 2015. Particle-associated and cell-free extracellular enzyme activity in relation to nutrient status of large tributaries of the Lower Mississippi River. Biogeochemistry. 124(1):255–271.](http://paperpile.com/b/eawJdl/z9Rg)

[Musonge PLS, Boets P, Lock K, Ambarita MND, Forio MAE, Goethals PLM. 2020. Rwenzori Score (RS): A Benthic Macroinvertebrate Index for Biomonitoring Rivers and Streams in the Rwenzori Region, Uganda. Sustain Sci Pract Policy. 12(24):10473.](http://paperpile.com/b/eawJdl/8rzK)

[Pimentão AR, Pascoal C, Castro BB, Cássio F. 2020. Fungistatic effect of agrochemical and pharmaceutical fungicides on non-target aquatic decomposers does not translate into decreased fungi- or invertebrate-mediated decomposition. Sci Total Environ. 712:135676.](http://paperpile.com/b/eawJdl/lwMp)

[Redondo-Hasselerharm PE, Falahudin D, Peeters ETHM, Koelmans AA. 2018. Microplastic effect thresholds for freshwater benthic macroinvertebrates. Environ Sci Technol. 52(4):2278–2286.](http://paperpile.com/b/eawJdl/pi4z)

[Rice EW, Baird RB, Eaton AD, editors. 2017. Standard Methods for the Examination of Water and Wastewater, 23rd Edition. American Public Health Association, American Water Works Association, Water Environment Federation.](http://paperpile.com/b/eawJdl/2DF7)

[Shirokova L, Vorobieva T, Zabelina S, Klimov S, Moreva O, Chupakov A, Makhnovich N, Gogolitsyn V, Sobko E, Shorina N, et al. 2016. Small Boreal Lake Ecosystem Evolution under the Influence of Natural and Anthropogenic Factors: Results of Multidisciplinary Long-Term Study. Water. 8(8):316.](http://paperpile.com/b/eawJdl/4nJk)

[Siddiqui E, Pandey J. 2019. Temporal and spatial variations in carbon and nutrient loads, ion chemistry and trophic status of the Ganga River: a watershed-scale study. Limnology . 20(3):255–266.](http://paperpile.com/b/eawJdl/4ry3)

[Simonin M, Colman BP, Anderson SM, King RS, Ruis MT, Avellan A, Bergemann CM, Perrotta BG, Geitner NK, Ho M, et al. 2018. Engineered nanoparticles interact with nutrients to intensify eutrophication in a wetland ecosystem experiment. Ecol Appl. 28(6):1435–1449.](http://paperpile.com/b/eawJdl/4xHk)

[Wang X, Zhang Y, Tan X, Zheng Y, Zhang Q. 2021. Do water quality, land use, or benthic diatoms drive macroinvertebrate functional feeding groups in a subtropical mountain stream? Inland Waters. 11(1):67–77.](http://paperpile.com/b/eawJdl/3E9f)

[Wilson CJ, Brain RA, Sanderson H, Johnson DJ, Bestari KT, Sibley PK, Solomon KR. 2004. Structural and functional responses of plankton to a mixture of four tetracyclines in aquatic microcosms. Environ Sci Technol. 38(23):6430–6439.](http://paperpile.com/b/eawJdl/YKvH)
